# Enhanced gain and detectivity of unipolar barrier solar blind avalanche photodetector via lattice and band engineering

**DOI:** 10.1038/s41467-023-36117-8

**Published:** 2023-01-26

**Authors:** Qingyi Zhang, Ning Li, Tao Zhang, Dianmeng Dong, Yongtao Yang, Yuehui Wang, Zhengang Dong, Jiaying Shen, Tianhong Zhou, Yuanlin Liang, Weihua Tang, Zhenping Wu, Yang Zhang, Jianhua Hao

**Affiliations:** 1 State Key Laboratory of Information Photonics and Optical Communications & School of Science, Beijing University of Posts and Telecommunications, Beijing, 100876 P. R. China; 2 Institute of Modern Optics & Tianjin Key Laboratory of Micro-Scale Optical Information Science and Technology, Nankai University, Tianjin, 300071 P. R. China; 3 Department of Applied Physics, The Hong Kong Polytechnic University, Hung Hom, Hong Kong, P. R. China; 4 The Hong Kong Polytechnic University Shenzhen Research Institute, Shenzhen, 518057 P. R. China

**Keywords:** Semiconductors, Sensors and biosensors

## Abstract

Ga_2_O_3_-based solar blind avalanche photodetectors exhibit low voltage operation, optical filter-free and monolithic integration of photodetector arrays, and therefore they are promising to be an alternative to the bulky and fragile photomultiplier tubes for weak signal detection in deep-ultraviolet region. Here, by deliberate lattice and band engineering, we construct an n-Barrier-n unipolar barrier avalanche photodetector consisting of *β*-Ga_2_O_3_/MgO/Nb:SrTiO_3_ heterostructure, in which the enlarged conduction band offsets fortify the reverse breakdown and suppress the dark current while the negligible valance band offsets faciliate minority carrier flow across the heterojunction. The developed devices exhibit record-high avalanche gain up to 5.9 × 10^5^ and detectivity of 2.33 × 10^16^ Jones among the reported wafer-scale grown Ga_2_O_3_-based photodetectors, which are even comparable to the commercial photomultiplier tubes. These findings provide insights into precise manipulation of band alignment in avalanche photodetectors, and also offer exciting opportunities for further developing high-performance Ga_2_O_3_-based electronics and optoelectronics.

## Abstract (web summary)

The limited breakdown electric field and the high dark current hider the application of solar blind avalanche photodiodes. Here, Zhang et al., by lattice and band engineering, construct a unipolar barrier avalanche photodiode with suppressed dark current and reinforced reverse breakdown.

## Introduction

Solar blind photodetectors (PDs) enable photoelectric conversion within the wavelengths of 200–280 nm. They generate electrical signals solely responding to deep-ultraviolet (DUV) light, with particular applications ranging from security communication to fire alarm detections^1–3^. To date, solar blind detection in a wide range of applications has been mainly accomplished by photomultiplier tubes (PMTs) based on the non-thermionic vacuum tubes made of glass, which are favored in weak UV signal sensing resorting to their ultrahigh internal gain (~10^6^)^4,5^. However, PMTs operate at high voltage above 1 kV and need external Wood’s optical filters to eliminate the influence from longer wavelength light, the attendant bulky power sources with strict requirements of high stability, evacuated glass construction, magnetic shielding requested in some circumstances, and fragile optical filter. Putting all these limitations of PMTs together, it is imperative to explore alternatives to PMTs, and all-solid-state PDs are ideal candidates in principle by considering their potential of overcoming the PMTs’ problems and capabilities of future monolithic integration. Wide-bandgap semiconductors such as AlGaN, MgZnO, and Ga_2_O_3_ have sprung to the forefront of solar blind detection activity owing to their key attributes, such as intrinsic solar rejection, high breakdown electric field, high chemical and thermal stability^6–8^. In contrast to PMTs, wide-bandgap semiconductor-based solar blind PDs may enjoy the features of low-voltage operation and optical filter-free integration, and promise more compact and robust monolithic integration of PD arrays. To meet the requirements of the detector’s cutoff wavelength of 280 nm, alloying engineering is commonly implemented to achieve high Al/Mg contents in AlGaN and MgZnO compounds. Unfortunately, owing to the high growth temperature (*T* > 1350 °C) for AlGaN and wurtzite–rocksalt phase segregation in MgZnO, plenty of defects/dislocations occurred in ternary semiconductor AlGaN and MgZnO films, inevitably degrading the relevant devices’ performances^9,10^. Inspiringly, Ga_2_O_3_ with a suitable bandgap (~4.9 eV) has been recognized as an excellent candidate suitable for promoting solar blind PDs^11–14^. In the past decade, Ga_2_O_3_-based solar blind PDs with different architectures and designs have been proposed, including photoconductive PDs^15–17^, Schottky barrier PDs^18,19^, and avalanche PDs (APDs)^20–22^. APDs promise orders of magnitude higher responsivity and gain than other types of PDs, and conceivably are becoming a research hotspot in this field^23^. Despite unremitting efforts, the performance metrics for the reported avalanche PDs (APDs) including responsivity and avalanche gain still lag behind the benchmark of commercial PMTs. The strategy for further enhancing the detectivity and internal gain of APDs heavily relies on increasing the reverse bias while blocking the dark current.

Due to the paucity of bipolar doping, and the complexity of heterojunction formation, only a handful of high-gain Ga_2_O_3_-based APDs have been proposed based on n–n isotype architecture^20–22^. Nevertheless, the conduction band offsets (Δ*E*_C_) are limited by the difference between the electron affinity *χ*_s_ of the constitutive n-type semiconductors. Further improvement in internal gain can be achieved by constructing Ga_2_O_3_-based heterojunctions with enlarged barrier height, or rather large Δ*E*_C_. Modification of the barrier height has been carried out by inserting a wide bandgap insulator barrier to form an n-Barrier-n (nBn) heterostructure, in which the enlarged band offsets fortify the reverse breakdown and suppress the dark current^24,25^. On further analysis, an nBn unipolar barrier design in APDs can significantly improve the performance of solar blind PDs. For Ga_2_O_3_-based APDs upon UV irradiation, photon-induced electrons and holes are accelerated and undergo cascade amplification through impact ionization with increasing the reverse bias. An appealing feature of unipolar barrier design is that a nearly zero valence band offset (Δ*E*_V_) allows the generated holes to flow through the barrier unimpededly, rather than accumulate at the interface occurred in conventional bipolar barrier structures. Unipolar barrier construction, however, suffers from the strict limitations associated with lattice and band matching. To date, unipolar barrier PDs have only been investigated in the visible and infrared region^24–27^.

The advent of synthesizing high-quality epitaxial Ga_2_O_3_ on oxides provides enticing opportunities to build an nBn unipolar barrier APDs with dramatically enhanced performance. The challenging issue for building Ga_2_O_3_-based heterojunctions is the intrinsic large bandgap of Ga_2_O_3_, which limits the selection of dielectrics as the designed barrier. The bandgap of MgO can reach up to 7.83 eV, which is much larger than that of Ga_2_O_3_ (4.94 eV), favoring the construction of a rectifying junction with a large band offset. Here, we construct unipolar barrier solar blind APDs consisting of *β*-Ga_2_O_3_/MgO/Nb:STO heterostructures. The deliberately designed heterostructure possesses the Δ*E*_C_ between *β*-Ga_2_O_3_/MgO and MgO/Nb:STO estimated up to 3.01 and 4.18 eV, respectively, plus a near zero valence band offset Δ*E*_*V*_ formed at the interfaces. The proposed unipolar barrier APDs exhibit recording-high gain and ultrahigh detectivity among the reported wafer-scale grown Ga_2_O_3_-based PDs, which are comparable with commercial PMTs. The work presents an unexploited architecture of unipolar barrier design applied in solar blind APDs, and the proposed strategies have tremendous potential to develop Ga_2_O_3_-based electronics requiring high breakdown fields.

## Results

### Device fabrication and characterization of epitaxial heterostructures

**Fig. 1:**
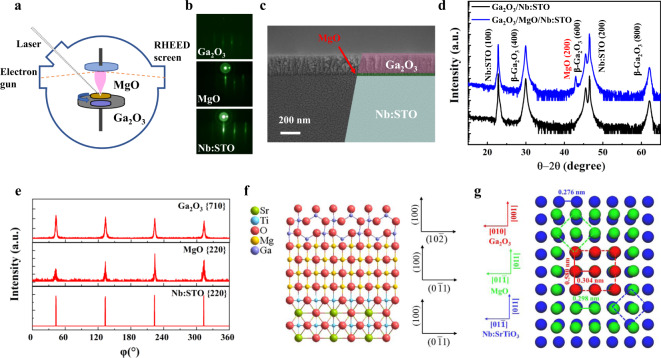
Synthesis and structural characterizations of the nBn unipolar barrier heterostructure. **a** Schematic process for heterostructure growth. **b** Reflection high-energy electron diffraction (RHEED) patterns of Ga_2_O_3_, MgO, and Nb:STO. **c** Cross-sectional SEM image. **d** XRD *θ*−2*θ* scan. **e** XRD Φ-scan. **f** Schematic atomic arrangement of the cross-sectional heterostructure. **g** Schematic interfacial oxygen-atom arrangements.

To design an effective unipolar barrier APD device, both interfacial lattice compatibility and energy band offsets need careful consideration. By taking account of our previous experimental results^28^ (Supplementary Fig. S1), MgO was incorporated with Ga_2_O_3_ to form a unipolar barrier structure possessing a large conduction band offset Δ*E*_C_ and nearly zero valance band offset Δ*E*_V_. We fabricated an nBn unipolar barrier APD based on the as-prepared Ga_2_O_3_/MgO/Nb:STO heterostructure using laser molecular beam epitaxy (LMBE) equipped with in-situ reflection high-energy electron diffraction (RHEED) (Fig. 1a, growth details in the “Methods” section). A Ga_2_O_3_/Nb:STO heterostructure was also fabricated for the performance comparison with APD devices. The evolution of the RHEED specular spot intensity enables us to monitor the quality and thickness of both MgO and Ga_2_O_3_ films at the atomic scale. Figure 1b shows the typical RHEED patterns for a Ga_2_O_3_/MgO/Nb:STO heterostructure. The streaky RHEED patterns verify the flat interface and surface of the heterostructures when the depositions for both MgO and Ga_2_O_3_ films are completed. Atomic force microscopy (AFM) indeed reveals a surface topography with a root-mean-square (rms) roughness value <1.3 nm, as shown in Supplementary Fig. S2. Such a uniform and well-defined interface could minimize the interfacial defects/dislocations and therefore benefit the carrier flowing across the heterostructure. The thickness of Ga_2_O_3_ (200 nm) and MgO (25 nm) was confirmed by both ellipsometer and cross-sectional scanning electron microscopy (Fig. 1c). The X-ray diffraction (XRD) *θ−*2*θ* scan manifests sharp (*l*00) diffraction peaks of *β*-Ga_2_O_3_ and MgO, indicating that both Ga_2_O_3_/MgO/Nb:STO and Ga_2_O_3_/Nb:STO heterostructures are of the epitaxial form (Fig. 1d). And the out-of-plane relationship is *β*-Ga_2_O_3_[600]//MgO[200]//Nb:STO[200]. Off-specular Φ-scan was then conducted to investigate the in-plane film-substrate alignment. As shown in Fig. 1e, the oblique Ga_2_O_3_ $\left\{710\right\}$, MgO $\left\{220\right\}$ and Nb:STO $\left\{220\right\}$ Bragg reflections appear at coinciding angles and are separated azimuthally by 90°, revealing that the Ga_2_O_3_ lattice is rotated by 45° in contrast to MgO (100) surface. Thus, the in-plane relationship of the unipolar heterostructure could be assigned to *β*-Ga_2_O_3_[001]//MgO[011]//Nb:STO[011]. For monoclinic structured *β*-Ga_2_O_3_, the oxygen atoms along [010] and [001] directions are arranged approximately as equilateral squares, with a spacing of 0.304 and 0.29 nm, respectively^29^. On the other hand, the oxygen atoms spacing along [011] and [01${\bar{1}}$] directions in cubic structured MgO and Nb:STO have the same squared arrangement with a spacing of 0.298 and 0.276 nm, respectively. Therefore, when the Ga_2_O_3_/MgO/Nb:STO nBn unipolar heterostructure is constructed, Ga atoms in the *β*-Ga_2_O_3_ (100) plane could bond to the oxygen atom layer in the MgO/Nb:STO (100) plane with a 45° rotation. A schematic atomic picture of the cross-sectional heterostructure and the interfacial oxygen-atom arrangements are depicted in Fig. 1f, g, respectively. Thus, the exquisite control offered by the LMBE using in-situ RHEED monitoring of thin-film growth has enabled the direct integration of epitaxial Ga_2_O_3_/MgO heterostructure on Nb:STO.

### Unipolar barrier calculations

**Fig. 2:**
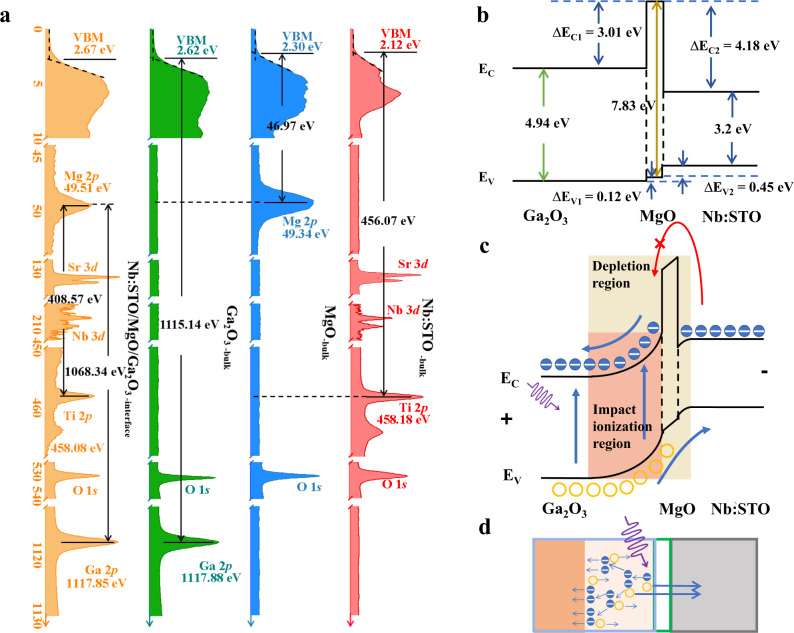
XPS characterization and band diagrams of the nBn unipolar barrier heterostructure. **a** Valence band maximum (VBM) spectra and the core levels for the Nb:STO bulk, MgO bulk, Ga_2_O_3_ bulk, and Ga_2_O_3_/MgO/Nb:STO interface. Band diagram of Ga_2_O_3_/MgO/Nb:STO heterostructure in equilibrium conditions (**b**) and in avalanche condition (**c**). **d** Illustration of the avalanche process in the nBn unipolar barrier APDs.

We conducted X-ray photoelectron spectroscopy (XPS) to explore the band diagrams of the Ga_2_O_3_/MgO/Nb:STO and the Ga_2_O_3_/Nb:STO heterostructures (Fig. 2a and Supplementary Fig. S3)^30^. According to Kraut’s method, the Δ*E*_V_ could then be determined by analyzing the binding energy difference between the valence band maximum (VBM) and the core-level shifts (see “Methods” section)^28^. The obtained Δ*E*_V_ for the Ga_2_O_3_/Nb:STO is 0.46 eV, while the Δ*E*_V_ for the Ga_2_O_3_/MgO/Nb:STO is $\triangle {E}_{{\rm {V}}-{\rm {Ga}}_{2}{\rm {O}}_{3}/{\rm {MgO}}}$ = 0.12 eV and $\triangle {E}_{{{{{{\rm{{V}}}}}}}-{{{{{\rm{MgO}}}}}}/{{{{{\rm{Nb}}}}}}:{{{{{\rm{STO}}}}}}}$ = 0.45 eV, respectively. Then, considering the bandgap (*E*_g_) values for Ga_2_O_3_ (4.94 eV), MgO (7.83 eV), and Nb:STO (3.2 eV), the calculated conduction-band offset (Δ*E*_C_) for the Ga_2_O_3_/Nb:STO is 1.28 eV. Importantly, the obtained valance band arrangements for Ga_2_O_3_/MgO/Nb:STO are $\triangle {E}_{{{{{{\rm{C}}}}}}-{{{{{\rm{Ga}}}}}}_{2}{{{{{\rm{O}}}}}}_{3}/{{{{{\rm{MgO}}}}}}}$ = 3.01 eV and $\triangle {E}_{{{{{{\rm{C}}}}}}-{{{{{\rm{MgO}}}}}}/{{{{{\rm{Nb}}}}}}:{{{{{\rm{STO}}}}}}}$ = 4.18 eV, respectively. Therefore, the band diagrams for both Ga_2_O_3_/MgO/Nb:STO and Ga_2_O_3_/Nb:STO heterostructures under equilibrium conditions are illustrated in Fig. 2b and Supplementary Fig. S4. With an elaborate design, the as-prepared nBn-type heterojunction exhibits desired unipolar barrier characteristics, i.e., a large Δ*E*_C_ and a negligible Δ*E*_V_ across the Ga_2_O_3_/MgO/Nb:STO heterointerfaces. The carrier transport behaviors of the heterostructures are controlled by the energy band structure. The general purpose of unipolar barrier design is blocking one type of carrier while allowing the flow of the other. In the case of APDs presented here, the large Δ*E*_C_ can effectively block the electron transfer from Nb:STO to Ga_2_O_3_, which is able to solve the bottleneck of dark current and further improve the performance of APDs. APDs work under a high reverse bias and therefore avalanche multiplication successfully occurs as designed when the excess carriers are formed upon the UV photon impact. The band alignment facilitates the separation and migration of photoexcited carriers. With increasing the reverse bias, photon-induced charge carriers are accelerated and undergo cascade amplification through impact ionization within the depletion layer. Ionization coefficients of both electrons and holes increase along with the electric field in the depletion region. Therefore, higher reverse voltage generally results in higher gain. The large breakdown electric field (~8 MV/cm) endows Ga_2_O_3_ a born figure-of-merit (FOM) as an excellent potential material for APDs. However, so far the breakdown electric field of reported Ga_2_O_3_-based APDs is limited by the barrier height of the rectifying junction, which is much less than the often quoted theoretical breakdown field of Ga_2_O_3_. Very recently, we constructed amorphous Ga_2_O_3_/ITO APDs with an enlarged barrier height (~2.07 eV), resulting in dramatically improved reverse bias voltage and photoresponse^21^. Herein, the enlarged Δ*E*_C_ between MgO and Nb:STO is further estimated to be as high as 4.18 eV, promising larger tolerance of higher reverse bias. Next, we consider the distinctive role of unipolar barrier design influencing the avalanche progress under working conditions (Fig. 2c). Note that because the electron concentration in Nb:STO (~10^20^ cm^−3^) is much larger than that in unintentional doped Ga_2_O_3_ (10^16^–10^17^ cm^−3^), the depletion region is mainly penetrated in Ga_2_O_3_ side. Upon light irradiation, photoexcited carriers are accelerated with increasing the reverse bias, and undergo the avalanche process in the depletion region (Fig. 2d). Under avalanche breakdown, the extensive multiplied electrons are mainly generated in Ga_2_O_3_ side, which is swept to the anode upon reverse bias, while a negligible Δ*E*_V_ across the heterojunctions facilitates the generated holes flowing unimpeded. In the meantime, the photogenerated electrons in Nb:STO side are still blocked by the barrier. Electrons and holes should be separated in the ideal APDs as efficiently as possible, with minimal relaxation of charge carriers from the avalanche multiplication. Unipolar barrier construction filters out photocurrent components on demand, rather than aggregating them at the interfaces, which can reduce the adverse recombination rate. Furthermore, the hole aggregation would cause the built-in electric field to counteract the externally applied field, and impair the avalanche process. To sum up, the dark electrons mainly originated from Nb:STO side is suppressed by the large band offset in the conduction band, while the photocurrent arises from the Ga_2_O_3_ side. The enlarged conduction band barrier suppresses the dark current and promotes the avalanche gain owing to the enhanced breakdown field.

### Unipolar barrier APDs

**Fig. 3:**
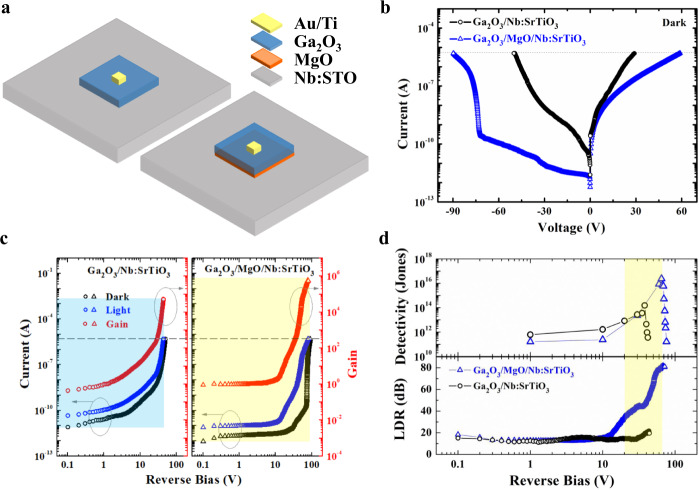
APDs performance comparison of the nBn unipolar barrier heterostructure and the n–n isotype heterostructure. **a** Schematic device illustration of the Ga_2_O_3_/MgO/Nb:STO nBn unipolar barrier APD, and the Ga_2_O_3_/Nb:STO n–n isotype APD. b Current–voltage (*I*–*V*) characteristics of both devices. **c** Reverse *I*–*V* curves in dark and under UV illumination for both devices; the right axis illustrates the gain. **d** Detectivity and LDR as a function of reverse bias for both devices.

Figure 3a schematically presents the device of Ga_2_O_3_/MgO/Nb:STO nBn unipolar barrier APD and Ga_2_O_3_/Nb:STO n–n isotype APD. As the nBn unipolar barrier heterostructure is designed to suppress dark current and enhance avalanche gain, carrier transport behaviors in dark were investigated, as shown in Fig. 3b. To prevent an unrecoverable breakdown in both APDs, 5 μA corresponding to the current density of 500 μA/mm^2^ is set as the limit current. Obvious rectifying behaviors are observed in the current–voltage (*I*–*V*) curves of both two types of heterostructures. In consistence with the proposed transport mechanism as discussed above, the dark current in the nBn unipolar barrier heterostructure is much smaller than that in the n–n isotype heterostructure, especially under a reverse bias. Note that the dark current in the Ga_2_O_3_/MgO/Nb:STO heterostructure remained below 0.06 nA (0.006 μA/mm^2^) under 50 V reverse bias, while the dark current in the Ga_2_O_3_/Nb:STO already reached 5 μA (500 μA/mm^2^). Thus, the large conduction band barrier exhibits significant inhibition of dark current over more than one order of magnitude, which provides great advantages for improving the internal gain and detectivity of APD. Figure 3c demonstrates the dark current and photocurrent (under light irradiation of 0.1 μW/cm^2^ at the wavelength of 254 nm), as well as the calculated gain *versus* reverse voltage for the two types of APDs. Here, the unmultiplied current at 1 V was assigned as the reference of unity gain. The unity responsivity is calculated by *R* = (*I*_Ph_−*I*_d_)/(*P* × *S)*, where *P*, *S*, *I*_Ph_, and *I*_d_ represent incident light intensity, effective irradiation area, photocurrent, and dark current, respectively^21^. Under the condition of 1 V bias and 0.1 µW/cm^2^ light irradiation, *I*_Ph_ is 9.8 pA, *I*_d_ is 2.2 pA, *P* is 0.1 µW/cm^2^, *S* is 10^4^ μm^2^ (the electrode area is ignored for convenience of calculation). Therefore, the *R* is calculated to be 0.76 A/W. It is found that the avalanche breakdown threshold voltages (calculated onset avalanche electric fields) are 31 V (1.55 MV/cm) and 71 V (3.16 MV/cm) for the Ga_2_O_3_/Nb:STO and the Ga_2_O_3_/MgO/Nb:STO heterostructures, respectively. The avalanche gain (*M*) is determined using the following relation: *M* = [*I*_Ph_(*V*)−*I*_d_(*V*)]/[*I*_Ph_(0)−*I*_d_(0)], where *I*_Ph_(*V*), *I*_d_(*V*), *I*_Ph_(0), and *I*_d_(0) are the multiplied photocurrent, dark current, unmultiplied photocurrent, and dark current, respectively^23^. Usually, the unity gain *M* = 1 is indicated when the photocurrent is almost constant for the low reverse bias regime. Thus, we take the unit gain at the voltage of 1 V. With increasing the reverse voltage, the avalanche gain values keep increasing exponentially, as shown in Fig. 3c (right axis). Compared to the maximum *M* value of 5.3 × 10^4^ at 43.6 V (2.18 MV/cm) in the Ga_2_O_3_/Nb:STO n–n isotype APD, the maximum *M* value for the Ga_2_O_3_/MgO/Nb:STO nBn unipolar barrier APD increases by one order of magnitude and reaches as high as 5.9 × 10^5^ at 78.1 V corresponding to electric field of 3.47 MV/cm, which is the record-high avalanche gain value among the reported Ga_2_O_3_-based solar-blind APDs. Considering the stability and reproducibility of the device, the working bias could be set at 73.2 V, where the gain can still reach as high as 4.4 × 10^5^. The dark current is limited to around 1 nA, while the photocurrent can reach up to 3.32 µA, more than three orders of magnitude of dark current. Another point worth noting is that the avalanche breakdown voltage of the junction determines the work voltage range of related devices. If we set the threshold of avalanche gain at 10^4^, the device’s operation voltage range of the nBn unipolar barrier heterostructure reaches up to ~30 V (from 50 to 80 V), which is much broader compared to the n-n isotype APD (~4 V) and other reported APDs (<5 V). Graphically, the “performance squareness” defined by three key parameters of APD including dark current, reverse working voltage, and gain is significantly amplified for unipolar barrier APD. Thus, significant improvements by the order of magnitude in both dark current and gain verify our unipolar barrier design as aforementioned. Investigation of the temperature-dependent threshold voltages presents a positive temperature coefficient of 0.026 V/°C, indicating that the underlying mechanism of the breakdown is attributed to the avalanche effect instead of the Zener tunneling effect (Supplementary Fig. S5). The avalanche gain *M* is mainly a function of the temperature. With the temperature increasing, the *M* is decreased under the identical reverse bias (Supplementary Fig. S6), due to the increased background dark current as well as the enhanced phonon scattering rate. To access the ultraweak light detection ability of the APD device, several critical FOMs, specific detectivity (D*), and linear dynamic range (LDR) were later quantitatively evaluated. The responsivity for both devices as a function of reverse bias is displayed in Supplementary Fig. S7. The current noise density spectra under various reverse biases are shown in Supplementary Fig. S8. Based on the measured current noise data and the responsivity results, the detectivity of the device could be calculated by D* = *R*(*S*Δ*f*)^1/2^/*i*_noise_, where *i*_noise_ is the noise current, Δ*f* is the electrical bandwidth (1 Hz)^31^. The linear dynamic range is calculated by LDR = 20 × log(*I*_Ph_/*I*_d_)^32^. As displayed in Fig. 3d, the maximum D* increases from 1.53 × 10^14^ to 2.33 × 10^16^ Jones, and the maximum LDR increases from 21.7 to 81.9 dB for Ga_2_O_3_/Nb:STO and Ga_2_O_3_/MgO/Nb:STO heterostructures, respectively. The enhancement of D* and LDR values indicate that the MgO layer introduced through the deliberate lattice and band engineering plays a major role in the Ga_2_O_3_/MgO/Nb:STO nBn unipolar barrier APDs, leading to improved device performances of detecting ultraweak light with low noise and linear responsivity for a wide range of light intensities^32^.

**Fig. 4:**
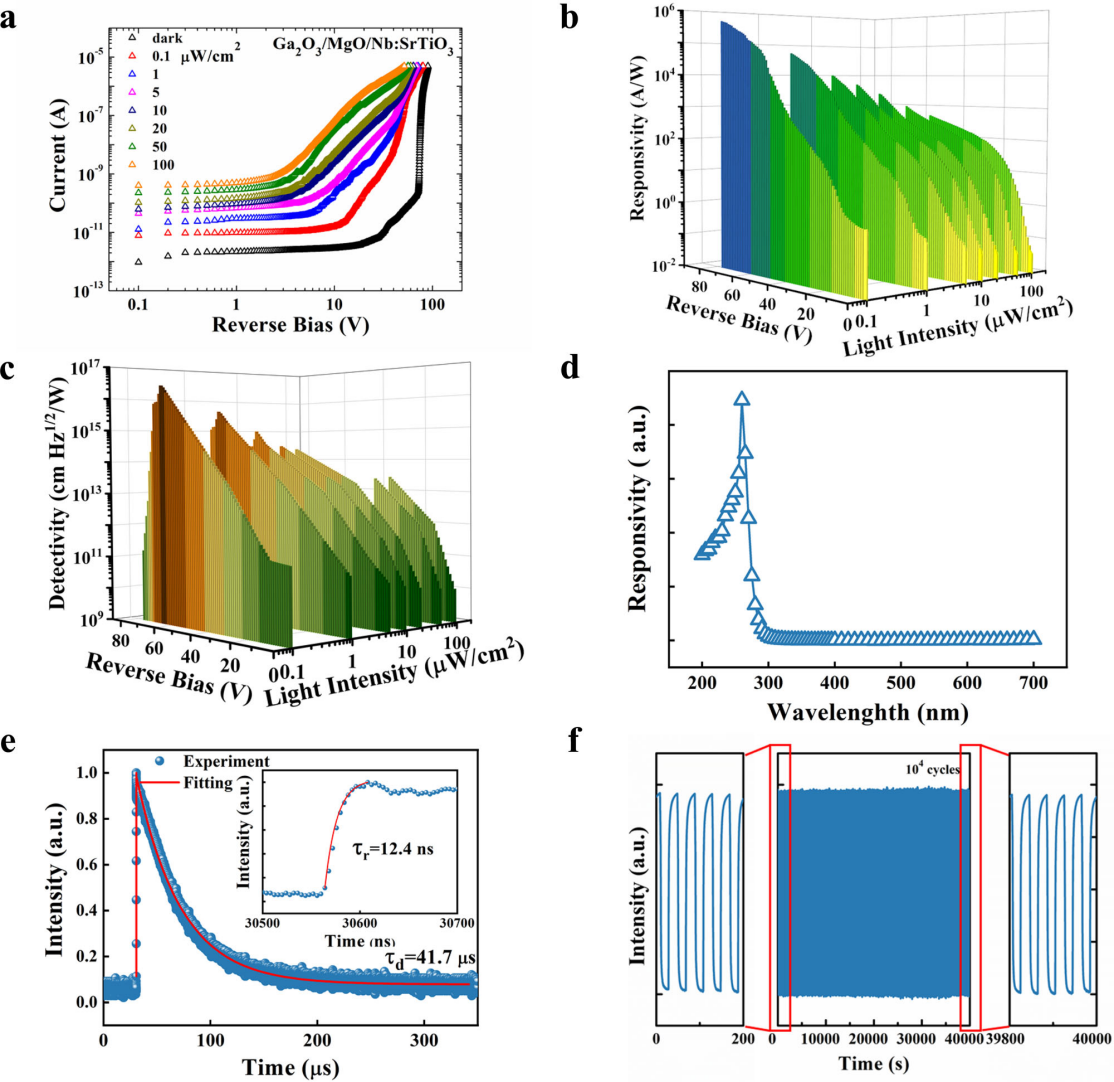
Photoresponse characteristics the nBn unipolar barrier APD. **a** Reverse *I*–*V* characteristics of the nBn unipolar barrier APD in the dark and under various intensities 254 nm light illumination. The responsivity (**b**) and detectivity (**c**) as functions of light intensities and applied bias. **d** Wavelength-dependent responsivity under reverse bias. **e** Transient response curve of the device; the inset reveals the enlarged rise edge. **f** Stability and reliability characteristics of the nBn unipolar barrier APD after 10^4^ light ON/OFF cycles.

The detailed photoresponse performance of the Ga_2_O_3_/MgO/Nb:STO nBn unipolar barrier APD was then systematically investigated. Figure 4a displays the *I*–*V* curves of the reverse-biased device measured under different light intensities, all revealing significant photoresponse behaviors. The *R* and D* as functions of light intensities and applied bias are presented in Fig. 4b, c, respectively. It is found that with an increase in light intensity, both *R* and D* of the device decrease. With increasing the incident light power, the number of free electrons increases and the carrier scattering rate raises. The increased number of free electrons tends to enhance the probability of electron–hole recombination, which may lead to the decreased trend of the *R* and D* at higher illumination^33^. Similar results were observed in many other types of photodetectors^19,34,35^. The responsivity can reach up to 4.46 × 10^5^ A/W under 0.1 μW/cm^2^ light irradiation at −78.1 V, which is among the best performance of Ga_2_O_3_-based solar-blind photodetectors^2,6,12^. The photoresponse performance of the Ga_2_O_3_/Nb:STO n–n isotype APD was also studied for comparison (Supplementary Figs. S9–S11). The spectral response of the device in the range of 200–700 nm was studied, as shown in Fig. 4d. The responsivity reaches its maximum value at ~260 nm with a cutoff edge at ~280 nm, revealing an excellent solar-blind region spectral selectivity. The response speed is another important parameter for the APD. To assess the temporal response speed of the APD, the transient photoresponse signal was measured using a coherent KrF 248 nm pulse laser as an illumination source along with a 500 MHz oscilloscope, as depicted in Fig. 4e. By fitting the transient response curves using $I={I}_{0}+{A}_{1}{{\rm {e}}}^{-t/{\tau }_{1}}+{A}_{2}{{\rm {e}}}^{-t/{\tau }_{2}}$ equation^6^, the obtained rise/decay time *τ*_r_/*τ*_d_ are 12.4 ns and 41.7 μs, respectively. One intuitive possible reason for such a slow response is the trap states due to the unintended defect as well as the surface states of Ga_2_O_3_. The traps states such as oxygen vacancies and/or dislocations may trap the photogenerated carriers in the device and prolong the lifetime of carriers, leading to a long response time for photodetectors. Thus, for improving the response speed, Ga_2_O_3_-based photodetectors are generally made into styles of heterostructures, Schottky diodes or APDs, where build-in fields are the driving force to separate photon-generated electron-hole pairs and promote photogenerated carriers transport. Although it still lags behind matured Si-based or InP-based PDs, the response speed of the nBn unipolar barrier APD developed in this work is already among the fastest Ga_2_O_3_-based PDs till now (Supplementary Table 1), which could be understood by the rapid electron–hole separation and transport owing to the enhanced avalanche electric fields and dispelling holes at the interface. The stability and reliability of the nBn unipolar barrier APD were further examined by periodically turning on/off the UV lamp. As displayed in Fig. 4f, the APD retained reliable photoresponse characteristics even after 10^4^ on/off cycles.

## Discussion

**Fig. 5:**
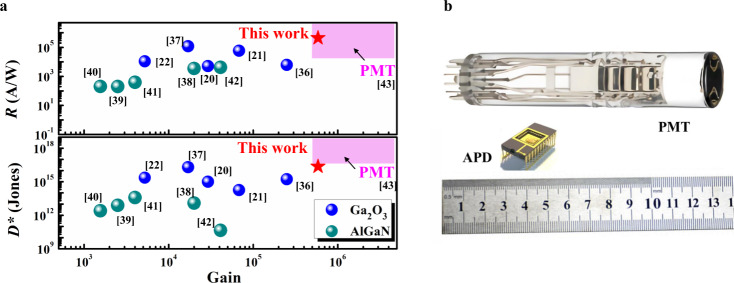
Performance comparison of the Ga_2_O_3_/MgO/Nb:STO nBn unipolar barrier APD with previously reported avalanche photodetectors (APDs) and photomultiplier tubes (PMTs). **a** Responsivity and detectivity versus gain. **b** Comparison of PMT and nBn unipolar barrier APD in terms of size, construction, and integration compatibility.

High dark current significantly impairs the performance of photodetectors. Unipolar barrier structures can suppress the dark current inside photodetector by blocking the majority of carriers. Constructing unipolar barrier photodetectors with conventional materials remains a daunting challenge, since the stringent requirements of lattice and band alignment. So far, the proposed unipolar barrier photodetectors are made of III–V AlGaAs or HgCdTe, since the lattice constant and bandgap can be tuned by the composition of semiconductors. Recently, Chen et al. devised an alternative unipolar barrier structure using van der Waals heterostructures, taking advantage of the assembly strategies regardless of lattice matching^24^. Due to the moderate bandgap of III–V and 2D materials, the reported unipolar barrier photodetectors can be operated in the visible and mid-wavelength infrared regimes. To date, there has yet to emerge a solution feasible for unipolar barrier photodetectors working in the ultraviolet regime, suffering from the limitations associated with the need for semiconductors endowed with suitable bandgap and the difficulty in heteroepitaxial growth. Herein, we constructed an n-Barrier-n (nBn) unipolar barrier APD consisting of *β*-Ga_2_O_3_/MgO/Nb:STO heterostructure to solve the problems. Ga atoms in the β-Ga_2_O_3_ (100) plane bonding to the oxygen atom in the MgO (100) plane with 45° in-plane rotation significantly alleviates the large lattice mismatch between them. The nBn heterostructure with a subtle selection of insulating dielectric MgO exhibits a large conduction band offset and near zero valence band offset. The deterministic configuration of the nBn unipolar barrier profile provides desired control over the charge carriers' flow and related avalanche multiplication effects. High dark currents are the primary contributor to noise and inherently limit the detectivity and gain in APDs. The enlarged conduction band barrier exhibits significant suppression of dark current over more than one order of magnitude. Moreover, the breakdown field predicted for the APD is determined by the rectifying junction with the barrier height. We have investigated the band alignment of Ga_2_O_3_/MgO/Nb:STO heterojunction with Δ*E*_C_ estimated up to 3.01 and 4.18 eV, which allows a maximum breakdown field (3.47 MV/cm) among all reported Ga_2_O_3_-based APDs, giving rise to a record-high avalanche gain value 5.9 × 10^5^ (at 78.1 V). In our case, the nBn unipolar barrier blocks the majority of electron carriers and allows the unimpeded flow of holes. Compared with bipolar barrier heterostructure, it reduces the recombination rate and facilitates the separation and transport of electrons and holes, leading to unprecedented APD characteristics, including gain, directivity and response speed. The main parameters for PDs are summarized in Supplementary Table 1, the responsivity and detectivity *versus* avalanche gain are also shown in Fig. 5a^20–22,36–43^. It is obvious that our developed APD demonstrates a record-high gain and FOMs according to its performance metrics, which is even comparable to commercial PMT. The comparison of PMT and nBn unipolar barrier APD in terms of size, construction, and integration compatibility is demonstrated in Fig. 5b^43,44^. More importantly, since Ga_2_O_3_ is emerging as a key building block for various applications of power electronics and next-generation optoelectronics, the tunable rectifying characteristics plus the integrating compatibility with dielectrics in this work offer exciting opportunities to build Ga_2_O_3_-based electronic and optoelectronic devices with dramatically improved performance.

## Methods

### Film growth and device fabrication

Commercial 10 × 10 mm^2^ (001)-oriented 0.05 wt% Nb-doped SrTiO_3_ (Nb:STO) single crystals (KJMTI) with a typical resistivity of 0.07 Ω cm were purchased as the substrates. 100 × 100 μm^2^ squared patterns were defined on the surface of Nb:STO substrates via standard photolithograhy, followed by a subsequent amorphous SrTiO_3_ deposition through a hard mask and lift-off processes. Next, a 25 nm-thick MgO barrier layer was grown on the Nb:STO substrate using laser molecular beam epitaxy (KrF 248 nm) with the following fabrication parameters: laser energy of 1.2 J/cm^2^, the pulse repetition rate of 1 Hz, substrate temperature of 700 °C and O_2_ pressure of 1 × 10^−2^ Pa. 200 nm-thick Ga_2_O_3_ film was subsequently grown on the MgO layer with the following fabrication parameters: laser energy of 0.8 J/cm^2^, the pulse repetition rate of 2 Hz, substrate temperature of 750 °C and O_2_ pressure of 1 Pa. A Ga_2_O_3_/Nb:STO sample (without MgO barrier layer) was also fabricated using the same parameter as a comparison. During the deposition, the growth of heterostructures was in situ monitored by RHEED to ensure precise atomic-level growth. After the growth, all samples were in-situ annealed in 200 mbar O_2_ at 470 °C for 30 min, before the final cooling down to room temperature. 30 × 30 μm^2^ squared Au/Ti electrodes (30 nm/20 nm) and gold pads (100 nm) were fabricated by sputtering on the top Ga_2_O_3_ layer and back side of the Nb:STO substrate, respectively, to form Ohmic contacts.

### Material characterizations and photoresponse measurements

X-ray diffractometer (Bruker D8 Discover: *λ* = 1.5406 Å, Cu Kα1 radiation) was employed to investigate the crystal structure of the samples. A Bruker DI3 atomic force microscope (AFM) was used to characterize the surface morphology of the samples. The X-ray photoelectron spectroscopy (XPS) measurements were carried out using a Thermo Fisher Scientific ESCALAB 250Xi instrument. The photoresponse *I*–*V* curves under varying irradiation intensities at the wavelength of 254 nm were recorded by a Keithly-4200 SCS semiconductor analyzer connected to a probe station using triaxial cables to ensure low-noise measurements. The noise spectral density characteristics were measured using FS380 Pro equipment (Platform Design Automation, Inc.), with the noise floor around 10^−28^ A^2^/Hz.

### Energy band alignments of the Ga_2_O_3_/MgO/Nb:STO heterostructure

XPS scan was used to quantitatively determine the energy band alignments of the nBn heterostructure. Four samples: Nb:STO substrate, MgO (100 nm)/Nb:STO, Ga_2_O_3_ (200 nm)/MgO (25 nm)/Nb:STO, and ultrathin Ga_2_O_3_ (3 nm)/MgO (2 nm)/Nb:STO were fabricated using the same growth condition described in the “Film growth and device fabrication” section. Kraut’s method was employed to calculate the valence band offset (Δ*E*_V_) and conduction band offset (Δ*E*_C_) by using the following equations:

1 $$\triangle {E}_{{{{{\rm{{V}}}}}}-{{{{{\rm{Ga}}}}}}_{2}{O}_{3}/{{{{{\rm{{MgO}}}}}}}}=\left({E}_{{{{{{\rm{{Ga}}}}}}-{{{{{\rm{core}}}}}}}}^{\rm {{Ga}}_{2}{\rm {{O}}}_{3}}-{E}_{{{\rm {VBM}}}}^{{{{\rm {Ga}}_{2}{O}}}_{3}}\right)-\left({E}_{{{\rm {Mg}-{{{{{\rm{core}}}}}}}}}^{{{\rm {MgO}}}}-{E}_{{{\rm {VBM}}}}^{{{\rm {MgO}}}}\right) \\ -\left({E}_{{{\rm {Ga}-{{{{{\rm{core}}}}}}}}}^{{{{\rm {Ga}}}}_{2}{\rm {{O}}}_{3}/{{\rm {MgO}}}/{{\rm {Nb}:{{{{{\rm{STO}}}}}}}}}-{E}_{{{\rm {Mg}-{{{{{\rm{core}}}}}}}}}^{{{{\rm {Ga}}}}_{2}{\rm {{O}}}_{3}/{{\rm {MgO}/{{{{{\rm{Nb}}}}}}:{{{{{\rm{STO}}}}}}}}}\right)$$

2 $$\triangle {E}_{{\rm {V-{MgO}}}/{\rm {{Nb}:{STO}}}}=\left({E}_{{\rm {{Mg}-{core}}}}^{{\rm {{Mgo}}}}-{E}_{{{\rm {VBM}}}}^{{{\rm {MgO}}}}\right)-\left({E}_{{{\rm {Ti}-{core}}}}^{{\rm {{Nb}:{STO}}}}-{E}_{{{\rm {VBM}}}}^{{{\rm {Nb}:{STO}}}}\right)\\ -\left({E}_{{{\rm {Mg}-{core}}}}^{{{{\rm {Ga}}}}_{2}{\rm {{O}}}_{3}/{{\rm {MgO}}}/{\rm {{Nb}:{STO}}}}-{E}_{{{\rm {Ti}-{core}}}}^{{{{\rm {Ga}}}}_{2}{{\rm {O}}}_{3}/{{\rm {MgO}/{Nb}}}:{{\rm {STO}}}}\right)$$

where ${E}_{{{\rm {Ga}-{core}}}}^{{{{\rm {Ga}}}}_{2}{{\rm {O}}}_{3}}$, ${E}_{{{\rm {Mg}-{core}}}}^{{\rm {{MgO}}}}$, ${E}_{{{\rm {Ti}-{core}}}}^{{{\rm {Nb}:{STO}}}}$, ${E}_{{{\rm {VBM}}}}^{{{\rm {MgO}}}}$, ${E}_{{{\rm {VBM}}}}^{{{{\rm {Ga}}}}_{2}{{\rm {O}}}_{3}}$, and ${E}_{{{\rm {VBM}}}}^{{{\rm {Nb}:{STO}}}}$ are the core levels of Ga 2*p*, Mg 2*p*, Ti 2*p*, and binding energy of the VBM for Ga_2_O_3_ (200 nm)/MgO (25 nm)/Nb:STO sample, MgO (100 nm)/Nb:STO sample and Nb:STO substrate, respectively. Therefore, the obtained Δ*E*_V_ for Ga_2_O_3_/MgO and MgO/Nb:STO are 0.12 and 0.45 eV, respectively. Given the respective bandgaps (*E*_g_) for *β*-Ga_2_O_3_ (4.94 eV), MgO (7.83 eV), and Nb:STO (3.2 eV), the conduction band minimum (CBM) for the heterostructure could be consequently determined. And the obtained values of Δ*E*_C_ for Ga_2_O_3_/MgO and MgO/Nb:STO are 3.01 and 4.18 eV, respectively. The negligible Δ*E*_V_ and large Δ*E*_C_ values for Ga_2_O_3_/MgO/Nb:STO indicates that an nBn unipolar barrier heterostructure is formed.

## Supplementary information

Supplementary Information

## Data Availability

All data supporting the results of this study are available in the manuscript or the supplementary information. Additional data are available from the corresponding author upon request.
