# Supplementary material for: Enhanced gain and detectivity of unipolar barrier solar blind avalanche photodetector via lattice and band engineering
Source: Nat Commun. 2023 Jan 26;14:418. doi: 10.1038/s41467-023-36117-8 (PMC9877014; doi:10.1038/s41467-023-36117-8)
Supplement: Supplementary file 1 — Supplementary Information [file 41467_2023_36117_MOESM1_ESM.pdf]

Supporting Information

Enhanced Gain and Detectivity of Unipolar Barrier Solar Blind Avalanche Photodetector *via* Lattice and Band Engineering

Qingyi Zhang,[1] Ning Li,[1] Tao Zhang,[1] Dianmeng Dong,[1] Yongtao Yang,[1] Yuehui Wang,[1] Zhengang Dong,[1] Jiaying Shen,[1] Tianhong Zhou,[2] Yuanlin Liang,[2] Weihua Tang,[1] Zhenping Wu,[1,*] Yang Zhang,[2,*] Jianhua Hao[3,4*]

[1] State Key Laboratory of Information Photonics and Optical Communications & School of Science, Beijing University of Posts and Telecommunications, Beijing 100876, P. R. China

[2] Institute of Modern Optics & Tianjin Key Laboratory of Micro-Scale Optical Information Science and Technology, Nankai University, Tianjin 300071, P. R. China

[3] Department of Applied Physics, The Hong Kong Polytechnic University, Hung Hom, Hong Kong, P. R. China

[4] The Hong Kong Polytechnic University Shenzhen Research Institute, Shenzhen 518057, P. R. China

[*] Corresponding author. Emails: zhenpingwu@bupt.edu.cn, yangzhang@nankai.edu.cn, and jh.hao@polyu.edu.hk.

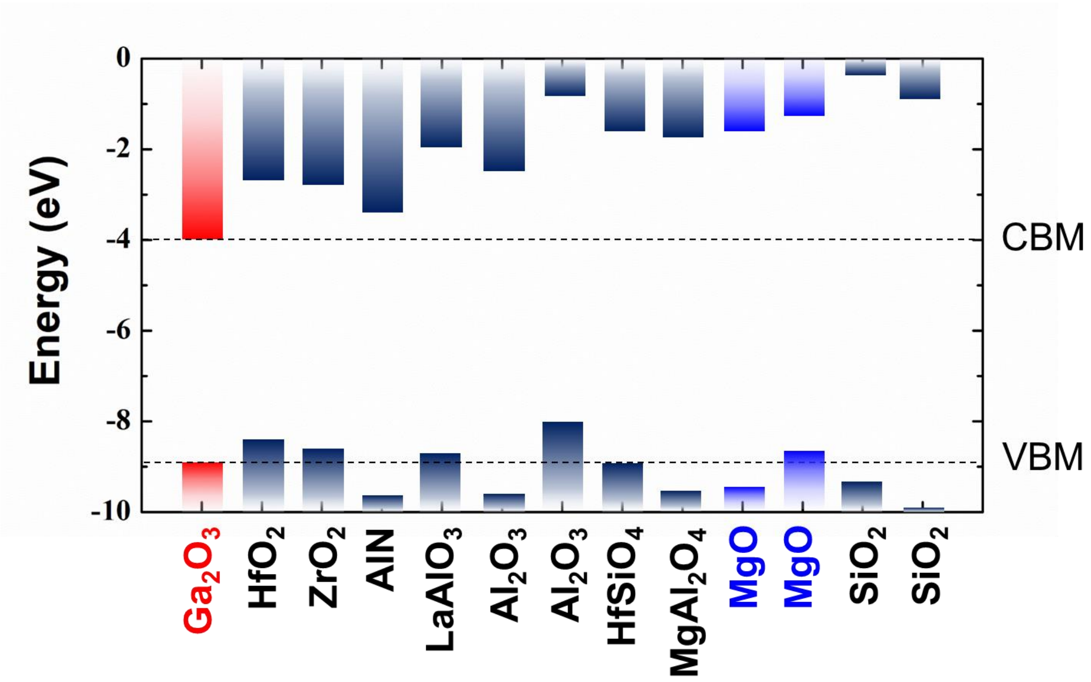

Fig. S1. Schematic band diagram of Ga_2O_3 with other wide bandgap dielectrics.

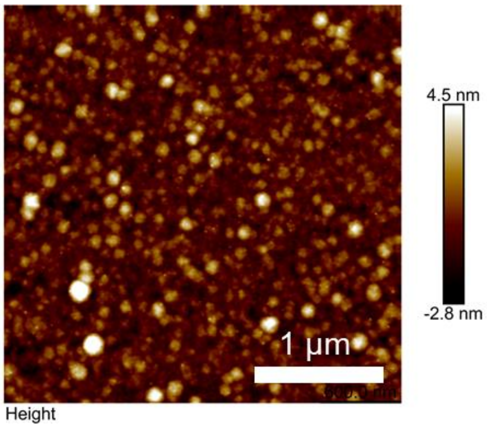

Fig. S2. Surface topography acquired by atomic force microscopy (AFM).

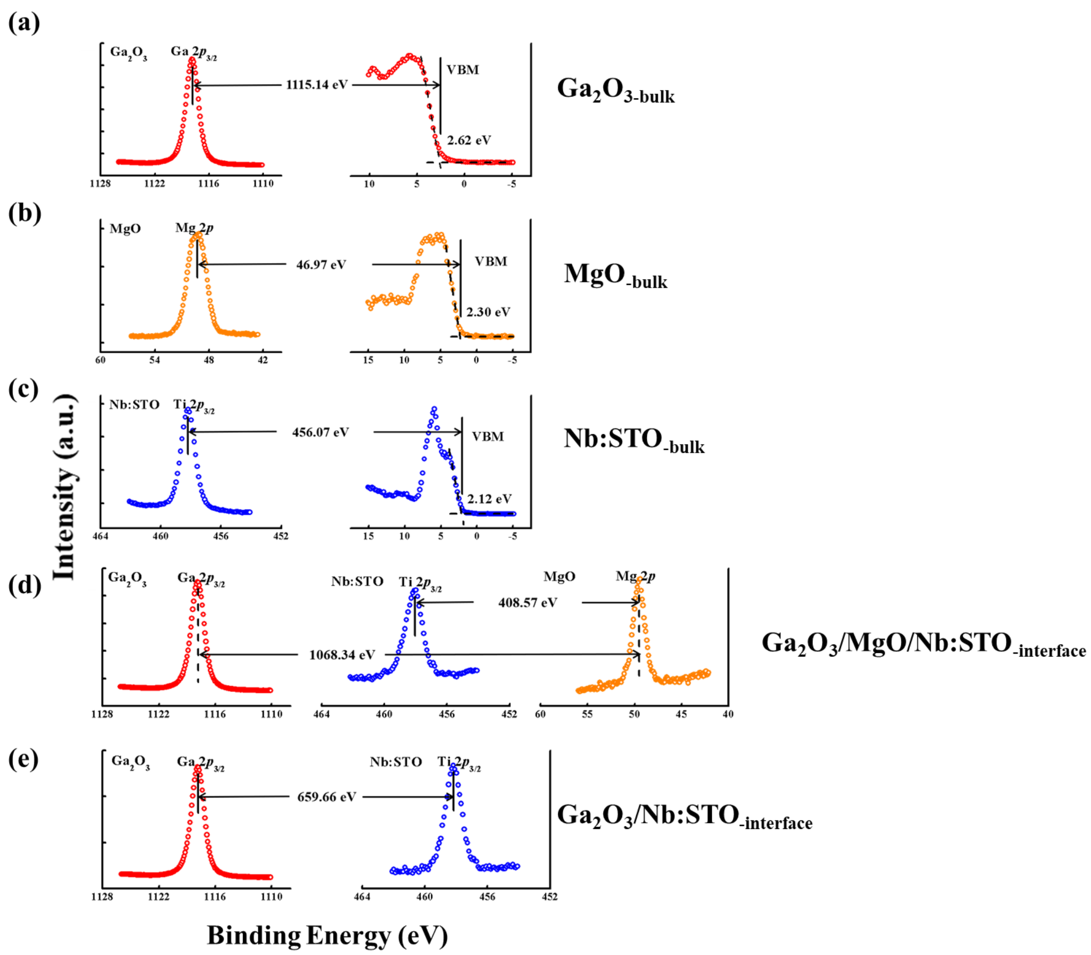

Fig. S3. XPS measurements of Ga_2O_3/MgO/Nb:STO and Ga_2O_3/Nb:STO heterostructures. (a) Ga 2p core level and valence band spectra of Ga_2O_3 bulk. (b) Mg 2p core level and valence band spectra of MgO bulk. (c) Ti 2p core level and valence band spectra of Nb:STO bulk. (d) Ga 2p, Mg 2p, and Ti 2p core-level spectra of the ultrathin Ga_2O_3 (3 nm)/MgO (2 nm)/Nb:STO. (e) Ga 2p and Ti 2p core-level spectra of the ultrathin Ga_2O_3 (3 nm)/Nb:STO.

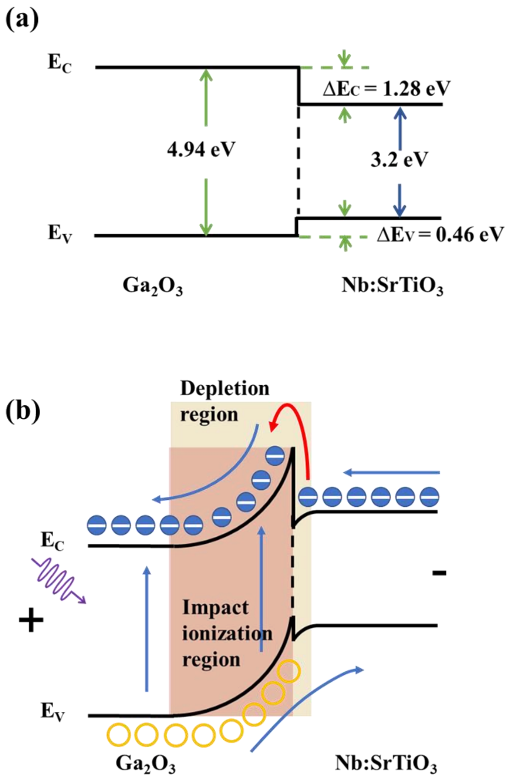

Fig. S4. Band diagram of Ga_2O_3/ Nb:STO heterostructure in equilibrium conditions (a) and in avalanche condition (b)

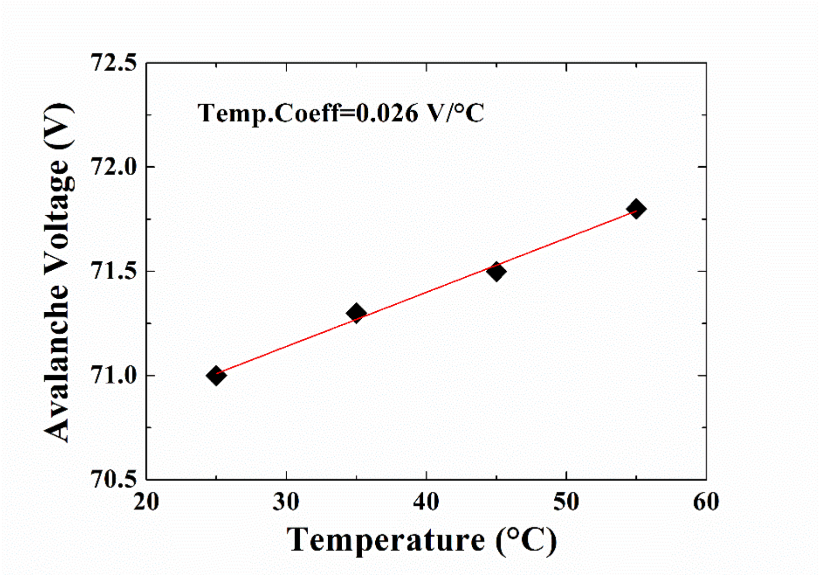

Fig. S5. Avalanche threshold voltage as a function of temperatures in dark.

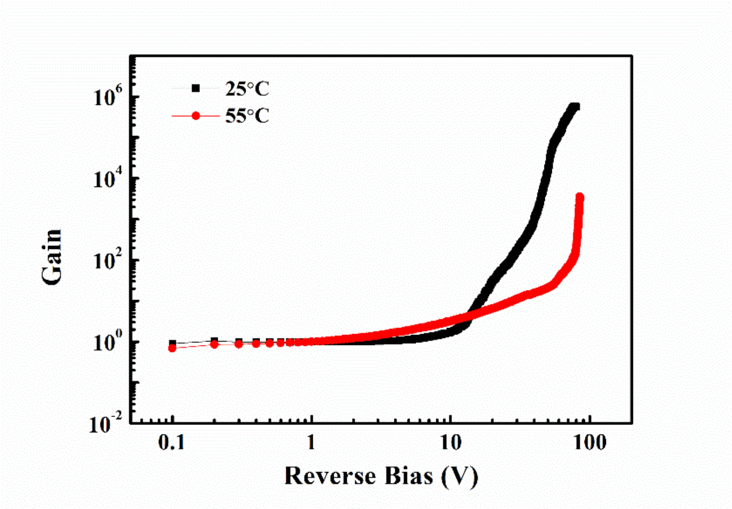

Fig. S6. Reverse bias dependence of Gain values at 25 °C and 55 °C.

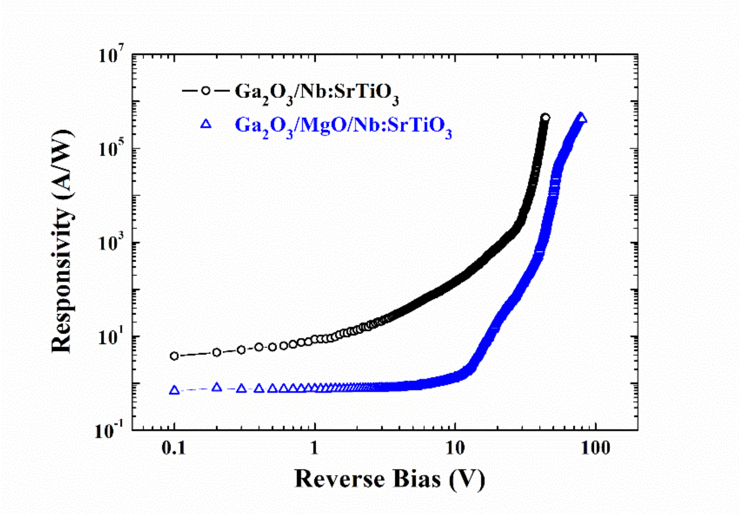

Fig. S7. Responsivity as a function of reverse bias for both nBn unipolar barrier heterostructure and the n-n isotype heterostructure.

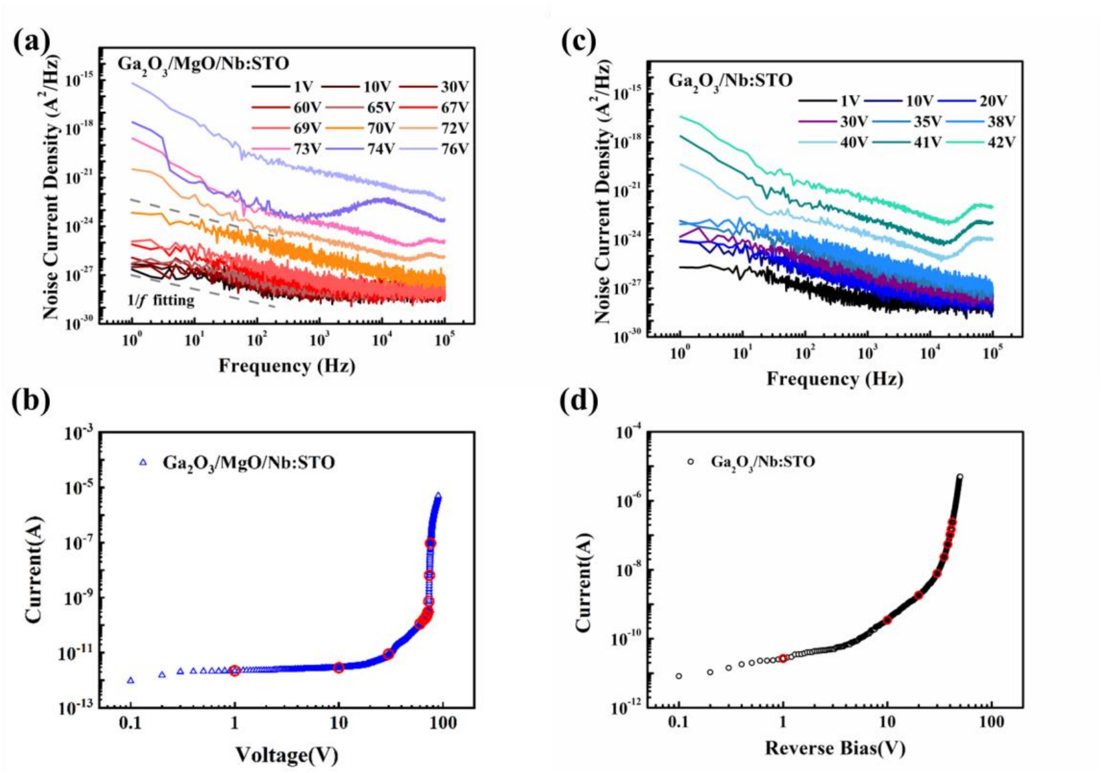

Fig. S8. (a) The current noise density spectra for Ga_2O_3/MgO/Nb:STO nBn unipolar barrier photodetector under various reverse biases. (b) The corresponding reverse bias points. (c) The current noise density spectra for Ga_2O_3/Nb:STO photodetector under various reverse biases. (d) The corresponding reverse bias points.

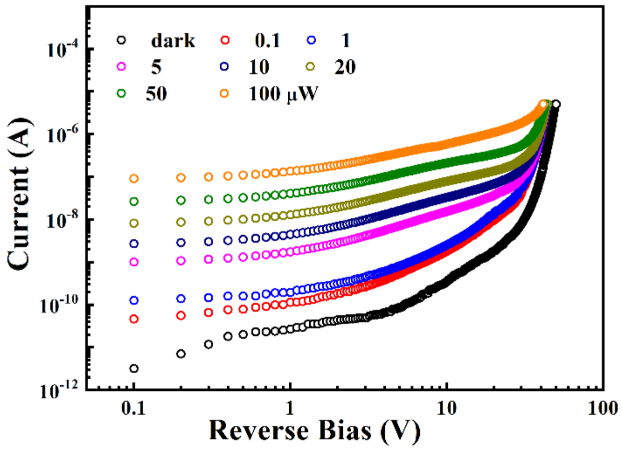

Fig. S9. Reverse *I-V* characteristics of the isotype Ga_2O_3/Nb:STO APD in the dark and under various intensity 254 nm light illumination.

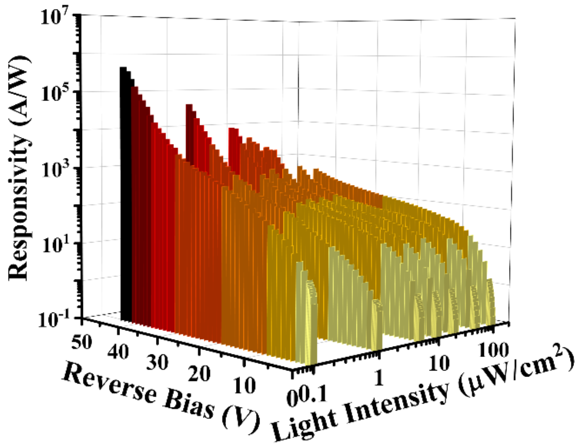

Fig. S10. The responsivity as functions of light intensities and applied bias of the isotype Ga_2O_3/Nb:STO APD.

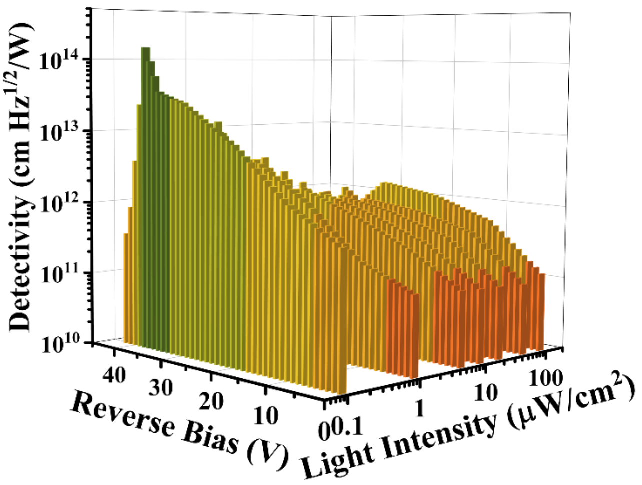

Fig. S11. The detectivity as functions of light intensities and applied bias of the isotype Ga_2O_3/Nb:STO APD.

Supplementary Table 1. Comparison of the figure-of-merits among reported Ga_2O_3 APD, AlGaN APD and PMT.

Device structures	Modes	Gain (a.u.)	R (A/W)	D* (Jones)	Bias (V)	Response speed	ref
ZnO−Ga_2O_3 Core−Shell	n-n	2.92×10^4	5.18×10^3	9.91×10^{14}	-10	20 μs/42 μs	[1]
β-Ga_2O_3/SnO_2	n-n	2.5×10^5	6.3×10^3	1.7×10^{15}	-10	48 μs/102 μs	[2]
ITO/Ga_2O_3	n-n	6.8×10^4	5.9×10^4	1.8×10^{14}	-40	-	[3]
α-Ga_2O_3/ZnO	n-n	5224	1.1×10^4	2.3×10^{15}	-40	238 μs/3.04 ms	[4]
Ga_2O_3	Schottky	1.7×10^4	1.2×10^5	2×10^{16}	-40	-/90 ms	[5]
AlGaN	p–i–n	2×10^4	3524	1.57×10^{13}	-140	-	[6]
AlGaN	p–i–n	2500	199.5	7.8×10^{12}	-62	-	[7]
AlGaN	Schottky	1560	202.8	2.54×10^{12}	-68	-	[8]
AlGaN	Schottky	4000	392	3.84×10^{13}	-177	-	[9]
AlGaN	p-i-n-i-n	4.12×10^4	4223	4.5×10^{10}	-108	-	[10]
PMT	-	$>5\times10^5$	$>1.4\times10^4$	$>5\times10^{16}$	-1500	4 ns/30 ns	[11]
Ga_2O_3/MgO/Nb:STO	nBn	5.9×10^5	4.46×10^5	2.33×10^{16}	-78.1	12.4 ns/41.7 μs	This work

Supplementary References

1 Zhao, B. *et al.* Solar-blind avalanche photodetector based on single ZnO-Ga_2O_3 core-shell microwire. *Nano Lett* **15**, 3988-3993, (2015).

2 Mahmoud, W. E. Solar blind avalanche photodetector based on the cation exchange growth of β-Ga_2O_3/Sno_2 bilayer heterostructure thin film. *Sol. Energy Mater. Sol. Cells* **152**, 65-72, (2016).

3 Wang, Y. *et al.* Ultrahigh gain solar blind avalanche photodetector using an amorphous Ga_2O_3-based heterojunction. *ACS Nano* **15**, 16654-16663, (2021).

4 Chen, X. *et al.* Solar-blind photodetector with high avalanche gains and bias-tunable detecting functionality based on metastable phase α-Ga_2O_3/ZnO isotype heterostructures. *ACS Appl. Mater. Interfaces* **9**, 36997-37005, (2017).

5 Li, Z. *et al.* High-performance β-Ga_2O_3 solar-blind schottky barrier photodiode with record detectivity and ultrahigh gain via carrier multiplication process. *IEEE Electron Device Lett.* **41**, 1794-1797, (2020).

6 Wu, H. *et al.* All AlGaN epitaxial structure solar-blind avalanche photodiodes with high efficiency and high gain. *Appl. Phys. Express* **9**, 052103, (2016).

7 Sun, L., Chen, J., Li, J. & Jiang, H. AlGaN solar-blind avalanche photodiodes with high multiplication gain. *Appl. Phys. Lett.* **97**, 191103, (2010).

8 Tut, T., Gokkavas, M., Inal, A. & Ozbay, E. $Al_xGa_{1-x}N$-based avalanche photodiodes with high reproducible avalanche gain. *Appl. Phys. Lett.* **90**, 163506, (2007).

9 Huang, Z., Li, J., Zhang, W. & Jiang, H. AlGaN solar-blind avalanche photodiodes with enhanced multiplication gain using back-illuminated structure. *Appl. Phys. Express* **6**, 054101, (2013).

10 Pau, J. L., Bayram, C., McClintock, R., Razeghi, M. & Silversmith, D. Back-illuminated separate absorption and multiplication GaN avalanche photodiodes. *Appl. Phys. Lett.* **92**, 101120, (2008).

11 *Photomultiplier tubes product from hamamatsu corp.*, <www.hamamatsu.com/us/en/product/optical-sensors/pmt/pmt_tube-alone/head-on-type.html> (2022).
